# Development of a High-Frequency, High-Temperature Class-A Amplifier Based on a Silicon Carbide Static Induction Transistor

**DOI:** 10.3390/s26123646

**Published:** 2026-06-08

**Authors:** Maximilian C. Scardelletti, Jonathon R. Grgat, Christian A. Zorman

**Affiliations:** 1 Communications and Intelligent Design Division at NASA Glenn Research Center, Cleveland, OH 44135, USA; 2 Department of Electrical, Computer and Systems Engineering, Case Western Reserve University, Cleveland, OH 44106, USA; jonathon.grgat2@gmail.com (J.R.G.); caz@case.edu (C.A.Z.)

**Keywords:** Class-A amplifier, silicon carbide, static induction transistor, high temperature, high frequency

## Abstract

**Highlights:**

**What are the main findings?**

- Development of a SiC SIT-based, Class-A amplifier for operation at a frequency of 50 MHz and a temperature of 400 °C.
- Development of an ADS-based circuit model to describe the high-temperature performance of the Class-A amplifier at high frequencies.

**What is the implication of the main finding?**

- Demonstrates the potential of a SiC SIT-based Class-A amplifier for high-temperature wireless sensor interface circuits.

**Abstract:**

This paper reports the development of a Class-A amplifier that operates at 50 MHz and 400 °C. The amplifier utilizes a commercially available 4H-SiC static induction transistor (SIT) as the active device and incorporates input/output-matching networks to optimize amplifier operation and DC bias networks, which comprise thin-film spiral inductors, metal–insulator–metal (MIM) capacitors, and thick-film chip resistors. All passive components were tested at frequency and temperature prior to amplifier development and are reported. A small-signal SiC SIT model that was developed in Keysight’s Advanced Design System (ADS 2023) software suite was used to design and optimize the amplifier’s performance. The SiC SIT amplifier’s S-parameters were recorded for frequencies between 20 and 100 MHz over a temperature range of 25 °C to 400 °C, exhibiting a gain (S_21_) of approximately 15.8 and 5.80 dB at 25 °C and 400 °C, respectively. The input and output reflection coefficients at 50 MHz and 400 °C were −18.5 and −15.2 dB, respectively. The noise figure and phase noise were measured at temperatures between 25 °C and 400 °C. At 50 MHz, the noise figure increased by only 21% over the temperature range, while the 1 kHz offset of the phase noise remained below −110 dBc/Hz. The stability factor, K, calculated using both measured and simulated data, demonstrates unconditional stability over the frequency range at 400 °C. Lastly, the 1 dB compression point was measured at 50 MHz and 400 °C with an approximated output of 9.5 dB. Simulated and measured results are presented and show the model is within 10% error at 400 °C.

## 1. Introduction

Health monitoring systems based on wireless sensors are in great demand to meet the technical requirements associated with next-generation gas turbine-based propulsion systems. Health monitoring systems that can ensure aircraft functionality and survival by detecting changes in emissions, temperature, blade tip clearance and pressure are under development [1,2,3,4,5,6,7]. Unfortunately, many of the best monitoring locations are positioned in or near the hot zones of the engine, requiring sensor systems that are compatible with extreme temperature, vibration, and pressure conditions. To complicate matters, the best locations for the sensors prohibit wired connections for power and data transfer, thus requiring the development of wireless sensor systems [8].

The preferred approach for such systems is one where the signal amplification and data transmission components are packaged along with the sensor in a single, miniaturized unit. Traditionally, silicon has been the semiconductor of choice for the active electronic components in wireless sensor systems, but with temperatures in the key locations of the gas turbine exceeding the range where silicon exhibits stable electrical properties [9], electronics made from wide-bandgap semiconductors such as silicon carbide (SiC) and gallium nitride (GaN) are being developed as alternatives to silicon for harsh environment applications.

Analog linear amplifiers are typically required to boost the transduced signal from the analog sensor or voltage-controlled oscillator (VCO). Moreover, in most cases, wireless microsystems require signal amplification of sensor output for wireless transmission. For gas turbine sensing applications, sensor systems must utilize a small form factor due to the tight tolerances associated with the engine. Because antenna size is inversely proportional to transmission frequency, wireless systems designed for space-constrained locations favor circuitry that operates in the radio frequency (RF) range to minimize antenna size for both the transmitter and receiver. SiC-based amplifiers have been successfully developed for high-temperature applications but unfortunately, these amplifiers operate at frequencies below what is needed for the aforementioned gas turbine engine applications. For example, a 6H-SiC op-amp designed to operate at 300 °C exhibited a voltage gain of 80 dB at low frequency and unity gain at 571 kHz [10]. A differential amplifier based on an n-channel SiC JFET operating at 576 °C had a gain of ~70 dB and unity gain at 3 MHz [11]. Lastly, a SiC op-amp operating at 500 °C exhibited a peak gain of ~39 dB and unity gain at 4.36 MHz has been successfully developed [12]. An effort to develop high-frequency amplifiers based on GaN has yielded a Class-A that operates at 255 MHz but is limited to operating at temperatures up to 230 °C [13]. Next-generation wireless sensor systems for gas turbine engine applications would benefit from amplifier circuits with an operating frequency in the 10 to 100 MHz range while exhibiting stable operation at 400 °C.

This paper describes the development of a novel, Class-A amplifier for operation under high-frequency and high-temperature conditions without the need for active cooling. Utilizing a commercially available SiC static induction transistor (SiC SIT) as the active component and passive components suitable for high-temperature operation, this amplifier was specifically designed to operate at a frequency of 50 MHz and a temperature of 400 °C, putting it first-in-class with respect to the combination of frequency and temperature. The amplifier circuit presented in this paper capitalizes on previous work by our group to characterize the SiC SIT for low-power, high-frequency operation at temperatures up to 400 °C and leverages our effort to develop a signal model suitable for modeling-based design of high-temperature SiC SIT amplifier circuits [14].

## 2. SiC SIT-Based Class-A Amplifier Design

**Figure 1:**
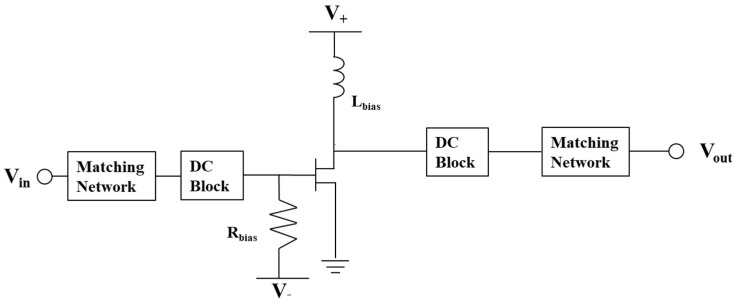
Block diagram of the SiC SIT-based Class-A amplifier.

A block diagram of the Class-A amplifier described in this paper is shown in Figure 1. The Class-A topology was chosen over other amplifier designs for several key reasons. Sensor applications that utilize high data rates require amplifiers with high linearity to process signals in real time, and in this regard, the Class-A amplifier is the most suitable design option for amplification of sensor output. The Class-A amplifier also ensures that important data is not lost by providing 360 degrees of conduction. Moreover, the simplicity of the design reduces the likelihood of component failure when operating in extreme-temperature environments by requiring the fewest number of components compared with comparable amplifier designs. Although efficiency may be increased by using other amplifier designs, the additional components or pulsed power associated with these designs may result in unwanted circuit degradation or catastrophic component failure during high-temperature operation.

**Figure 2:**
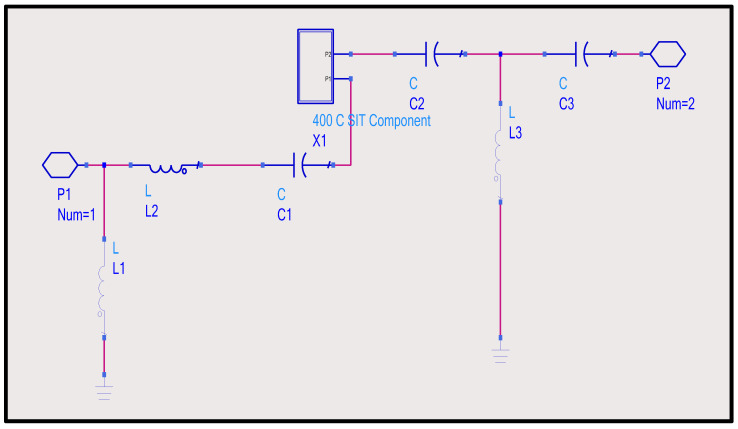
Circuit diagram of the SiC SIT-based Class-A amplifier rendered in ADS.

Advanced Design System (ADS) (Keysight, Santa Rosa, CA, USA) modeling software was used to simulate the amplifier circuit under high-temperature operation in order to select key parameters of the passive components based on the desired performance characteristics of the amplifier [15]. Figure 2 is a schematic diagram of the simulated amplifier circuit. A recent publication by our group describes an ADS-based small-signal model for the SiC SIT that accurately describes transistor performance up to 400 °C and frequencies between 20 and 100 MHz [14], and this model was integrated directly into the amplifier model. As reported in Ref. [14], a drain current of 40 mA and a drain voltage of 7 V were shown to be sufficient to achieve adequate transistor gain over the entire temperature range. A primary focus of the amplifier model was to identify the component parameters for the matching network to achieve high gain and low input/output reflections. In the simulations, the input frequency was swept from 20 MHz to 100 MHz to fully encompass an operating frequency of 50 MHz. Parameter values for the passive components were initially chosen by network calculations and then optimized by ADS through iterative solutions. The software employed a gradient optimization tool that paired with modeling objectives such as a maximum S_21_ gain at a given frequency or minimum values for the input/output reflections at a range of frequencies. Although the ADS optimization tool is quite powerful, setting too many objectives risks yielding unrealistic values, and thus, the tool was used with discretion. After optimization, if further circuit adjustments were necessary, a tuning function was used for fine adjustment of component values. The amplifier model was optimized to achieve a peak gain of 10 dB and input/output reflection coefficients lower than −18 dB at an operational frequency of 50 MHz and a temperature of 400 °C while matching the input and output to 50 Ω. Optimization and tuning resulted in an optimized amplifier circuit that utilizes the component values for the matching network and bias circuits found in Table 1.

## 3. Component Evaluation

To achieve stable operation at high temperatures, the simulated amplifier was constructed using a ceramic substrate rated for high-temperature operation, a commercially available SiC SIT, and a set of thick-film chip resistors, Au-based metal–insulator–metal (MIM) capacitors, and thin-film Au spiral inductors. Unlike the SiC SIT (Microsemi^TM^, Chandler, AZ, USA) that was previously shown to exhibit suitable operation at temperatures up to 400 °C and frequencies between 20 and 100 MHz [14], the resistors, capacitors and inductors required component-level evaluation at high temperatures prior to use in the amplifier circuit. High-temperature testing was performed using a custom-built high-temperature probe station described in detail elsewhere [16].

- Metal–Insulator–Metal (MIM) Capacitors

**Figure 3:**
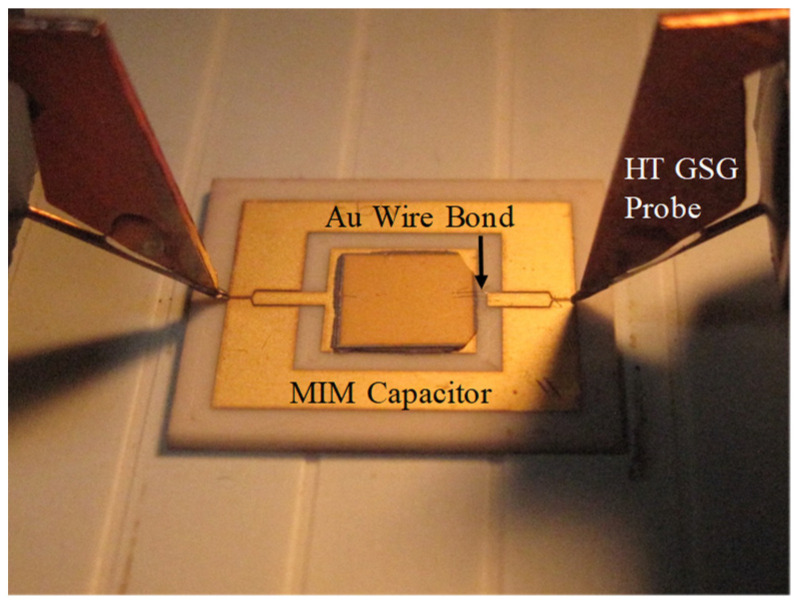
A photograph of a 5 mm^2^ (196 pF) test MIM capacitor mounted to a GSG test fixture for high-temperature testing.

MIM capacitors (M110 substrates, Maruwa Co. Ltd., Owariasahi, Aichi, Japan) with surface areas of 3.5 (97 pF) and 5 (196 pF) mm^2^ were used to test the insulating properties of the M110 substrates at temperatures between 25 °C and 400 °C. The M110 MIM capacitors had Ti/Au metallization for the top and bottom electrodes, an insulator with a relative permittivity of 110, and a substrate thickness of 0.127 mm. The capacitive surface areas of the MIM capacitors were chosen purely arbitrarily amongst other pre-cut MIMs with different surface areas to determine how the temperature influences the insulating properties of the dielectric. The capacitance was determined using the following equation:

(1) $$C=\frac{\varepsilon_{r}\varepsilon_{0}A}{d}$$

where *ε*_r_ is the relative permittivity of the substrate, *ε*_0_ is the permittivity of free space, *A* is the area of the overlapping top and bottom electrodes, and *d* is the distance between the electrodes. Capacitance values for the 3.5 and 5 mm^2^ capacitors recorded at 25 °C were 94 and 196 pF, respectively. To evaluate the performance of the capacitors as a function of temperature, high-temperature test fixtures made of 500 μm thick alumina substrates (CoorsTek 996 Alumina Superstrates, CoorsTek, Golden, CO, USA) coated with a ~10 μm electroplated Au layer were fabricated by laser micromachining (LPKF ProtoLaser, Garbsen, Germany). The laser was used to micromachine 150 µm pitch, ground–signal–ground (GSG) probe pads into the Au layer. High-temperature Ag epoxy was used to adhere a capacitor to a large, centrally located contact pad on the test structure, establishing electrical contact between the bottom electrode of the capacitor and metallization on the test fixture. A gold wire bond was used to make an electrical connection between the top electrode and the test fixture. Figure 3 is a photograph of a capacitor that has been mounted to a text fixture.

**Figure 4:**
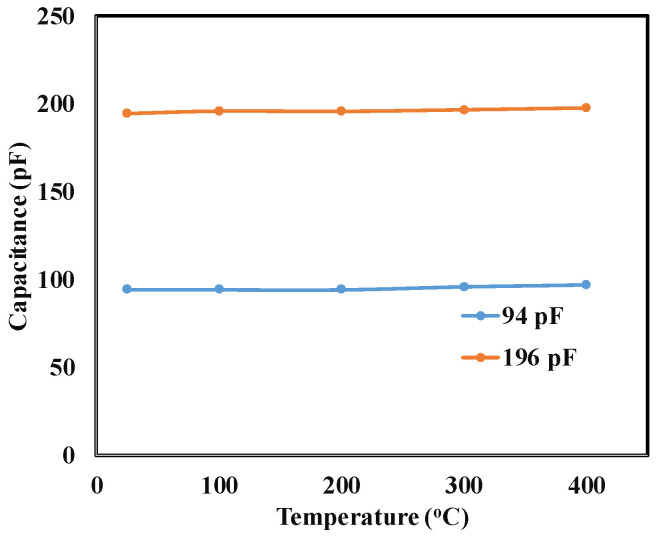
Representative C-V measurements for 94 and 196 pF MIM capacitors for temperatures between 25 °C and 400 °C. Measurements were performed at a frequency of 1 MHz.

DC high-temperature Au/W needle probes were used to make electrical connections from the GSG probe pads to the associated benchtop instruments via coaxial cables. All DC measurements were performed using a power analyzer (Keysight B1505A, Santa Rosa, CA, USA). Scattering parameters (S-parameters) were measured with a network analyzer (Agilent E836B, Santa Rosa, CA, USA) using the GSG 150 μm pitch high-temperature probes (GGB Industries Model P-12-9403, Naples, FL, USA) and standard RF cables. C-V measurements and S-parameter measurements were performed on each capacitor at five specific temperatures. C-V characteristics were measured using a power analyzer (Keysight B1505A) at 1 MHz with a bias voltage of 0 and 1 V. The measurements were performed on five capacitors of the same capacitance value to evaluate device-to-device variability. Figure 4 presents capacitance versus temperature plots from representative 94 and 196 pF capacitors. The 94 pF capacitor exhibited an increase in capacitance of 2.1%, while the increase in capacitance for the 196 pF capacitor was only 1.5%. These small increases are most likely due to the effects of temperature on the Ag epoxy and/or high-temperature probes and not to changes in the dielectric properties of the insulator.

**Figure 5:**
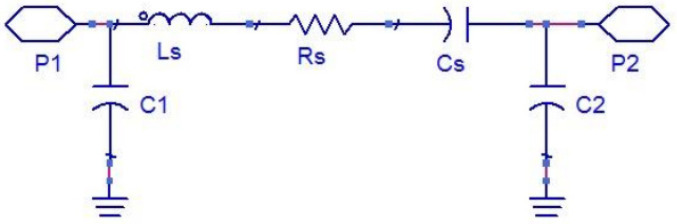
Circuit diagram of the lumped element model of the MIM capacitors.

**Figure 6:**
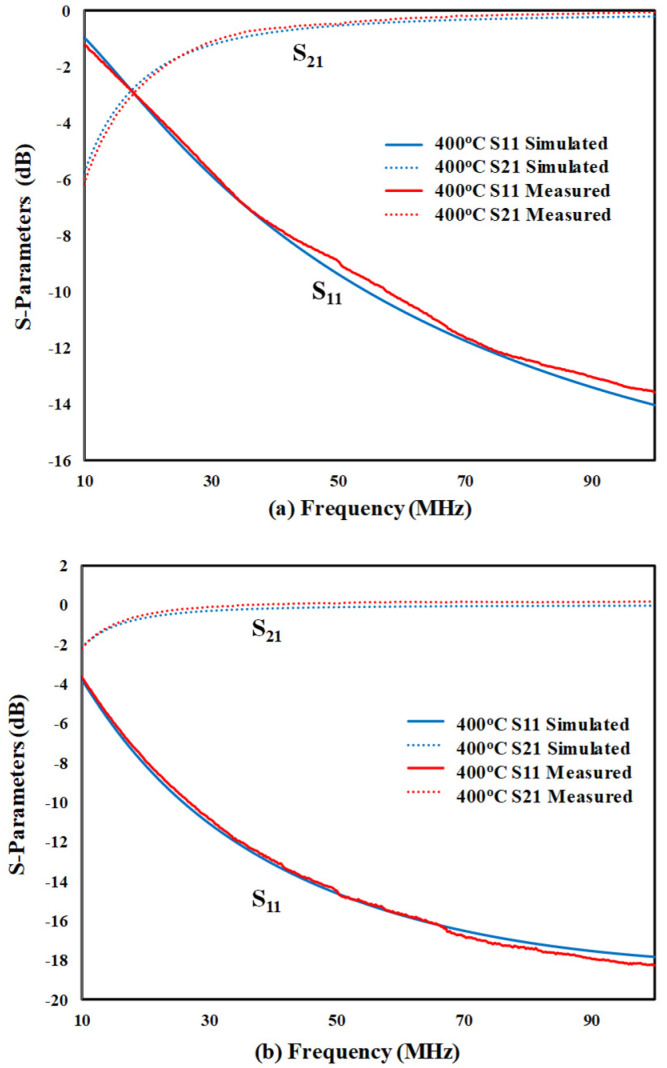
A comparison of measured and simulated S-parameters at 400 °C for: (**a**) a 94 pF capacitor and (**b**) a 196 pF capacitor.

S-parameter measurements were performed on the same set of capacitors over a frequency range from 10 to 100 MHz and a temperature range of 25 °C to 400 °C using a precision network analyzer (Keysight N5224B, Santa Rosa, CA, USA). To ensure accurate measurements, a short-open-load-through (SOLT) calibration was performed before each measurement for de-embedding cables, probe losses and placing the reference plane of the measurements at the probe tips. The measured S-parameters from 25 °C to 400 °C were compared to a corresponding simulated lumped element model in ADS and the component values in the model were extrapolated using an optimization method. The lumped element model consisted of a series capacitance, C_S_; parasitic capacitances, C_1_ and C_2_; a series resistance, R_S_; and a series inductance, L_S_, configured as shown in Figure 5. Figure 6 compares measured and simulated S_11_ and S_21_ parameters at 400 °C for the 94 and 196 pF capacitors and the list of the individual component values for the optimized lump-sum model for temperatures between 25 °C and 400 °C are shown in Table 2 and Table 3. Figure 6 shows strong agreement between measured and simulated values for S_11_ and S_21_ at 400 °C for both the 94 and 196 pF capacitors. The strong agreement between the simulated and measured results indicates that the capacitor lumped element model accurately represents the temperature-dependent insulting properties of the Maruwa MIM capacitors. These results show that the dielectric properties of the M110 insulator varies by less than 2% between 25 °C and 400 °C, indicating that the M110 insulator is well suited for high-temperature MIM capacitors.

B. Inductors

**Figure 7:**
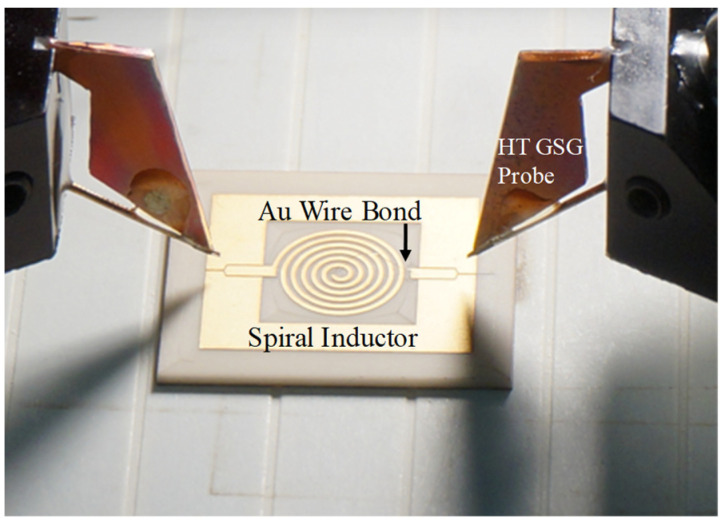
Photograph of a 94 nH thin-film inductor on the high-temperature probe station with GSG probes for S-parameter measurements.

**Figure 8:**
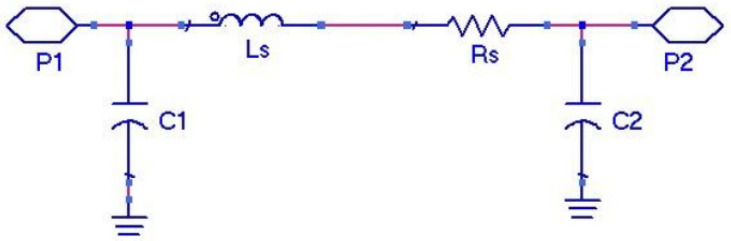
Circuit diagram of the lumped element model used to simulate the thin-film spiral inductors in ADS.

Thin-film spiral inductors were designed, fabricated and characterized from 25 °C to 400 °C and frequencies up to 100 MHz. The spiral inductors were designed using the electromagnetic simulation software, Sonnet™ 18.58, Syracuse, NY, USA., which accounts for material properties and dimensions to predict the inductance of a chosen design. The spiral inductors were fabricated by laser micromachining on alumina substrates (CoorsTek 996 Alumina Superstrates) that were coated with ~10 μm thick electroplated Au. Figure 7 is a photograph of a representative device under test, and Figure 8 presents the lumped circuit model used to simulate S-parameters in ADS.

**Figure 9:**
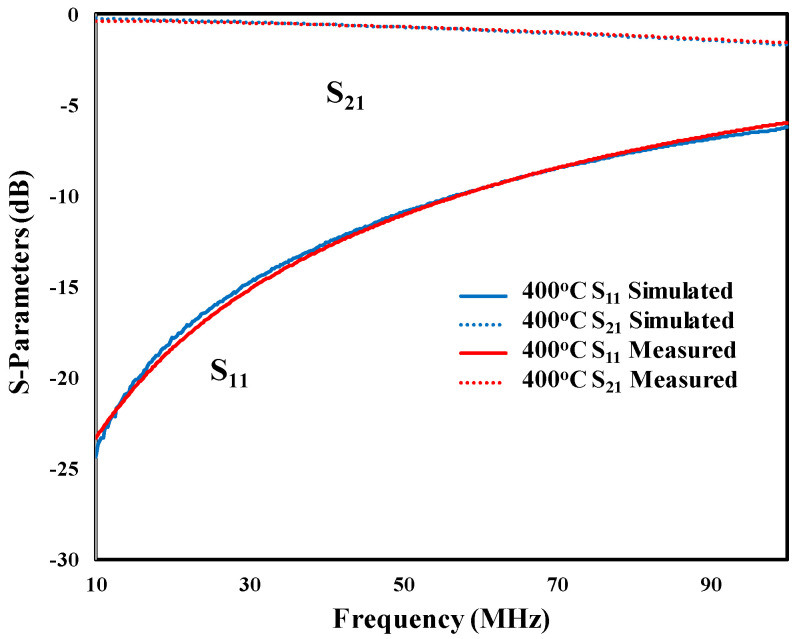
A comparison of measured and simulated S-parameters for a 94 nH spiral inductor at 400 °C.

S-parameter measurements were recorded from 10 to 100 MHz over a temperature range of 25 °C to 400 °C using a precision network analyzer. Following the same procedures as the MIM capacitors, optimization of the lumped element model was performed by comparing simulated values to the measured values as shown in Figure 9. Table 4 summarizes the component values for the lumped element model resulting from this optimization procedure. The optimized lumped element model indicates that the overall inductance increased by only 1.8% between 25 °C and 400 °C. In contrast, the parasitic series resistance increased by 244% between 25 °C and 400 °C due primarily to temperature-related losses in the metallization layer.

C. Resistors

**Figure 10:**
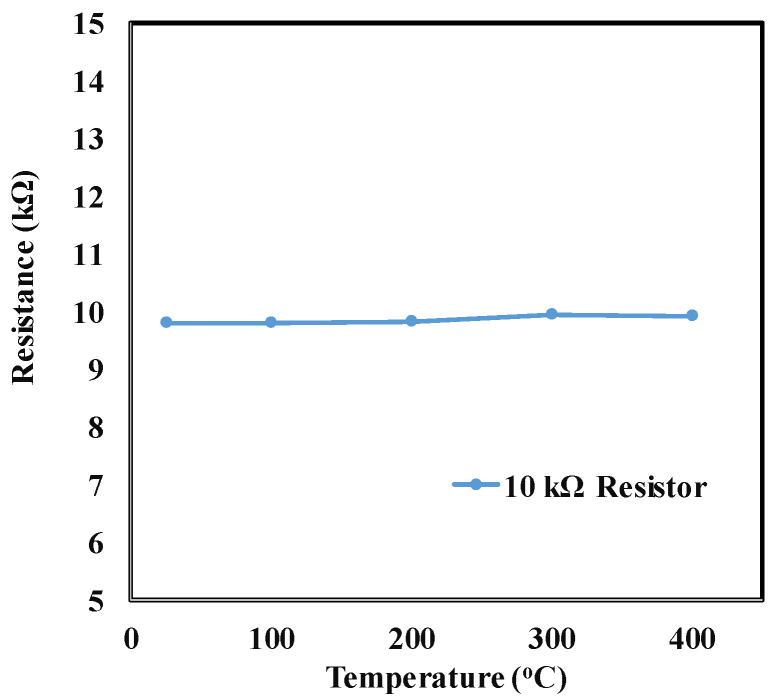
Resistance versus temperature for a representative 10 kΩ resistor.

A 10 kΩ thick-film chip resistor (MiniSystems Inc., Plainville, MA, USA) was utilized in the DC bias circuit at the gate of the SiC SIT to prevent RF from entering the gate side power supply. This 1.118 × 0.559 × 0.330 mm, thick-film chip resistor was constructed out of a ruthenium alloy with a voltage and power rating of 40 V and 1 W, respectively. High-temperature characterization was performed using a multimeter (Keysight 34401A, Santa Rosa, CA, USA), the Au/W needle probes, and the aforementioned high-temperature probe station. The measured resistance between 25 °C and 400 °C is plotted in Figure 10. The change in resistance over this temperature range is only 1.2%, indicating that this resistor is suitable for high-temperature use.

## 4. Fabrication of the Amplifier Circuit

**Figure 11:**
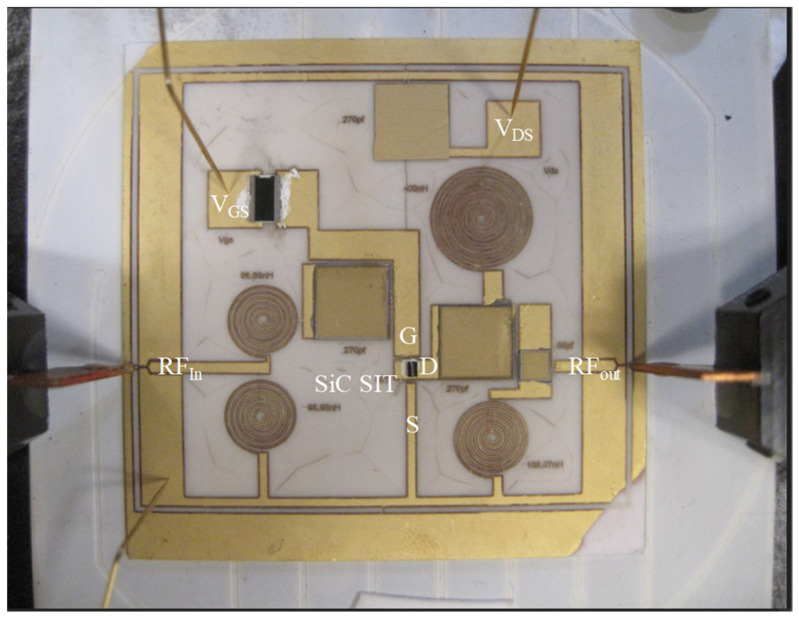
Optical photograph of the SiC SIT-based Class-A amplifier under testing conditions.

The SiC SIT amplifier was fabricated on a 50.8 × 50.8 mm alumina substrate (CoorsTek 996 Alumina Superstrate) coated with ~10 μm of electroplated Au. All the metal interconnects, contact pads and thin-film spiral inductors were fabricated out of the Au film by laser micromachining. Discrete components were adhered to the substrate using Ag epoxy, and Au wire bonds were used for electrical connections to contact pads on the substrate. A plan-view photograph of a fully fabricated amplifier is presented in Figure 11, highlighting the input (RF_in_) and output (RF_out_) ports; V_DS_ and V_GS_ DC probe pads; and the drain (D), gate (G), and source (S) pad locations of the SiC SIT. Figure 11 also shows the locations of the MIM capacitors, spiral inductors and thick-film resistors. RF measurements were performed using 150 μm pitch GSG high-temperature probes, while DC biasing utilized Au/W DC needle probes. The circuit was tested from 25 °C to 400 °C on a high-temperature probe station using a precision network analyzer (Keysight N5224B), signal analyzer (Agilent N9020A MXA), a signal generator (Agilent E8244A), and a power supply (Agilent 3649A, Santa Rosa, CA, USA).

## 5. Measurement Results

**Figure 12:**
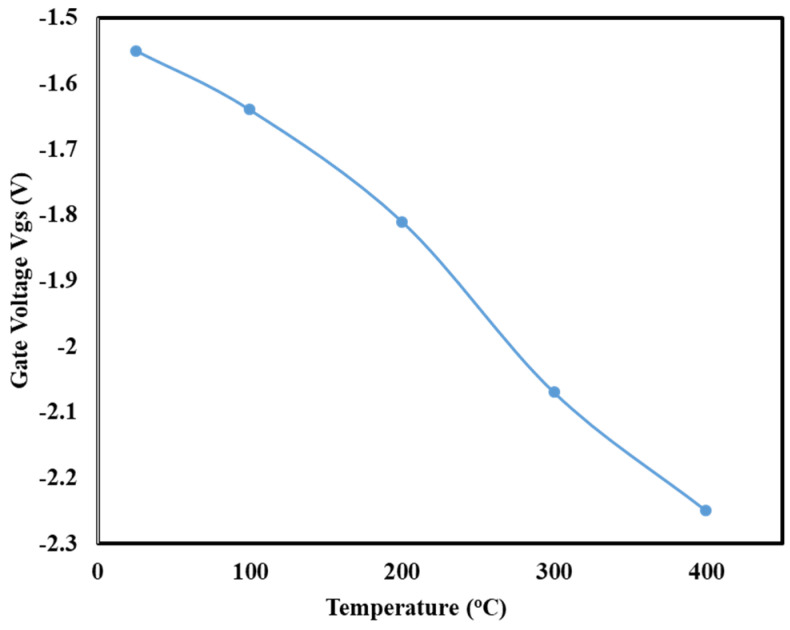
Gate voltage as a function of temperature under low-power operating conditions (I_DS_ = 40 mA and V_DS_ = 7 V).

Based on findings reported in Ref. [14], the drain current, I_ds_, for the SiC SIT was set at 40 mA to achieve suitable gain at a drain voltage, V_ds_, of 7 V, thus assuring low-power operation, which is required for stable operation at high temperatures. To achieve a constant current of 40 mA, a variable gate voltage, V_gs_, was required. As shown in Figure 12, the gate voltage decreased by roughly 40% from −1.56 V at 25 °C to −2.23 V at 400 °C. This reduction is because the current is modulated by the gate as well as the drain. At high temperatures, additional current is manifest due to the lowering of the potential barrier; a behavior that is characteristic of SITs [14].

**Figure 13:**
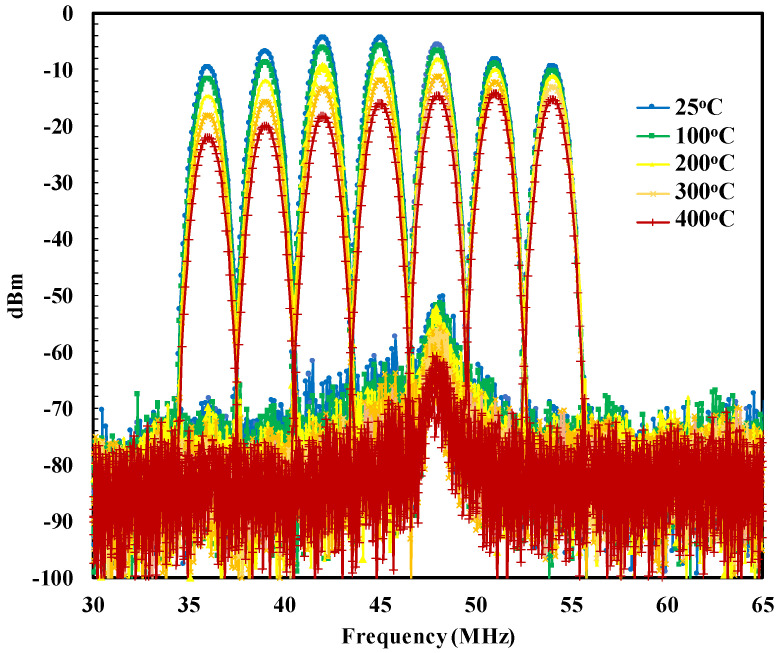
Output power versus frequency from 30 to 65 MHz for temperatures between 25 °C and 400 °C. The signal generator provided a −20 dBm single-tone to the input of the amplifier at frequencies between 36 and 54 MHz at increments of 3 MHz.

The output power as a function of temperature was measured between 36 and 54 MHz using a signal generator (Agilent E8244A, Santa Rosa, CA, USA) and a signal analyzer (Agilent N9020A MXA, Santa Rosa, CA, USA). The measurements were performed from 25 °C to 400 °C using the high-temperature probe station with the GSG probes at the RF input/output ports. The signal generator provided a −20 dBm single-tone signal to the input of the amplifier at frequencies of 36, 39, 42, 45, 48, 51, and 54 MHz. These frequencies were arbitrarily chosen to explore how temperature affects the power output envelope of the amplifier. The output was recorded with the signal analyzer and a LabView 2023 DAQ (Austin, Texas) program. The spectrum outputs for temperatures between 25 °C and 400 °C are shown in Figure 13.

As can be seen in this figure, the peak output power at 25 °C occurs at 42 MHz and is −4.20 dBm, which is a gain of 15.8 dBm. At 400 °C, the peak output power shifts to 51 MHz and is −14.2 dBm, corresponding to a gain of 5.80 dBm. The reason for the observed shift in peak-gain frequency is due to the temperature dependence of the parasitic elements in the SiC SIT model [14]. The transistor model used in the amplifier simulations was specific to a frequency of 50 MHz and a temperature of 400 °C and did not incorporate these known temperature-dependent behaviors at the lower temperatures. As a result, the parasitic element values for the SiC SIT used in the amplifier model are for 400 °C operation only. Previous work [14] demonstrates how the parasitic internal element values change as a function of temperature and, if not taken into account, would result in the observed shift in the peak-gain frequency. The loss in gain with increasing temperature can be attributed to a combination of effects including a decrease in transconductance and carrier mobility in the transistor as well as an increase in series resistances in the matching network.

**Figure 14:**
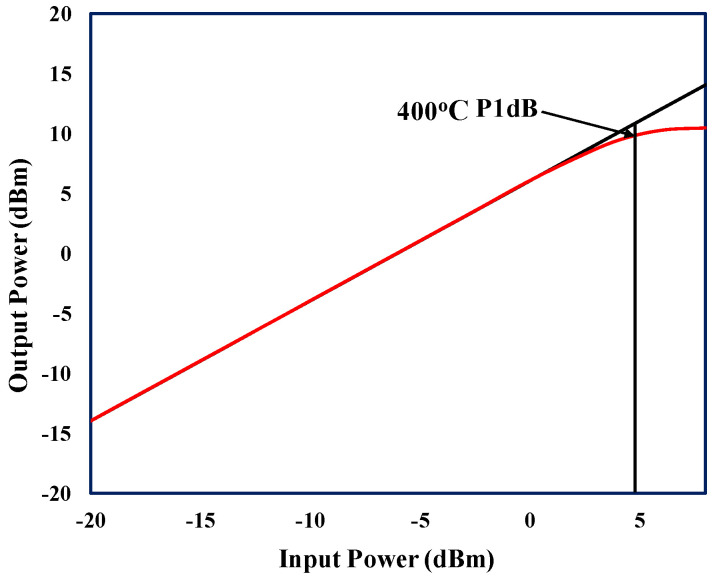
Measured output power versus input power at 400 °C.

Figure 14 shows the output power versus input power at 400 °C for an operating frequency of 50 MHz. The 1 dB compression (P1dB) point is defined as the position on the input power curve where the input power is below the expected linear extrapolation by 1 dB. Figure 14 indicates that the 1 dB compression point occurs at an input power of ~4 dB. The 1 dB compression indicates that the amplifier could operate as designed up to an input power of approximately 4 dBm at 400 °C

**Figure 15:**
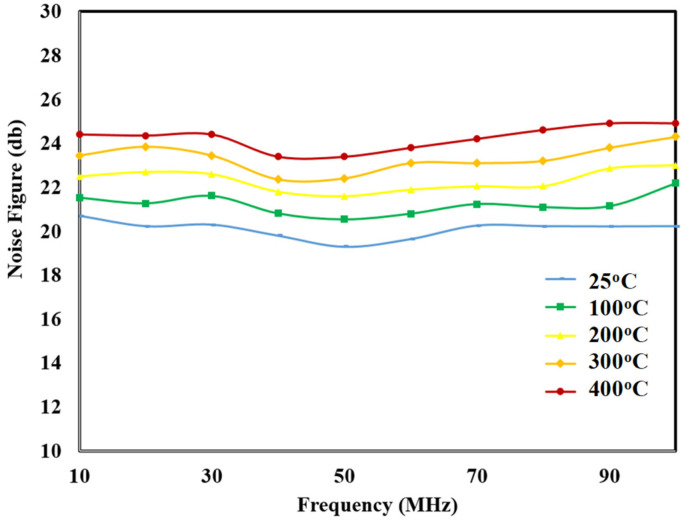
Measured noise figure (NF) as a function of frequency for temperatures between 25 °C and 400 °C.

The noise figure (NF) and phase noise were examined for the amplifier at temperatures between 25 °C and 400 °C. The NF was measured using a spectrum analyzer (Agilent E4448A, Santa Rosa, CA, USA) and a noise source (HP 346A, Santa Rosa, CA, USA) for frequencies between 10 and 100 MHz. The noise source was connected to the biased amplifier via RF in/out connections and the output was connected to the spectrum analyzer via high-temperature probes and RF cables. Biasing for the noise source was provided by the spectrum analyzer through BNC and coaxial cable connections. The noise source was calibrated to enable accurate measurement of the noise figure. Results of the NF measurement are presented in Figure 15. At each temperature, the NF is relatively stable across the frequency range. With regard to temperature, at a frequency of 50 MHz, the NF at 25 °C is 19.3, rising to 23.4 at 400 °C, an increase of only 21.2%. The observed increase in NF with increasing temperature is as expected

**Figure 16:**
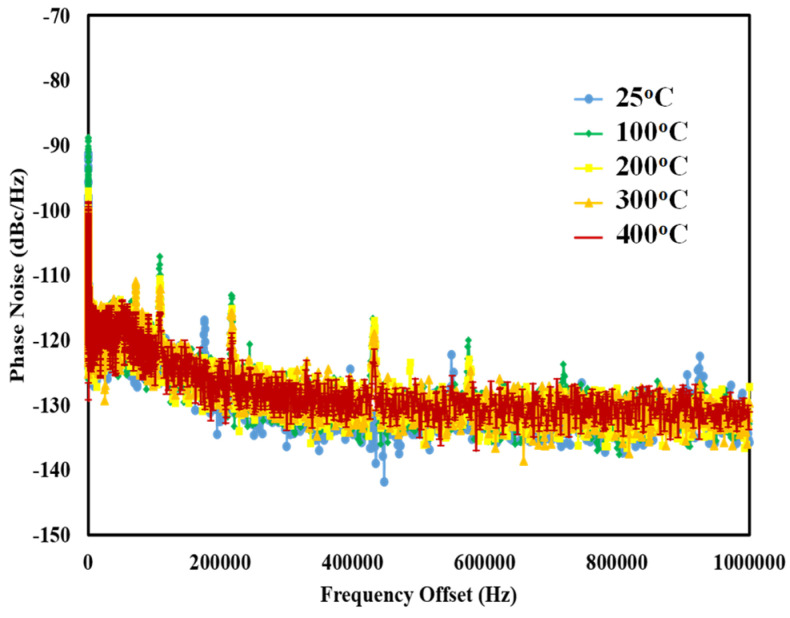
Measured phase noise versus frequency offset for temperatures between 25 °C and 400 °C.

The phase noise of the amplifier was measured using the series signal generator (Agilent E8244A) and the spectrum analyzer (Agilent E4448A) with built-in phase noise capability and RF in/out connections using the GSG high-temperature probes. A 50 MHz carrier signal from the signal generator was applied to the amplifier input, and the output was recorded on the spectrum analyzer. Figure 16 presents plots of phase noise versus frequency between DC and 100 MHz for temperatures between 25 °C and 400 °C. At all temperatures, the phase noise of the amplifier with respect to the input carrier signal at the 100 kHz offset frequency was less than −110 dBc/Hz. With respect to temperature, the phase noise at the 100 kHz frequency offset between 25 °C and 400 °C differed by less than 1%. This data shows that the amplifier does not add any appreciable phase noise to the input signal.

**Figure 17:**
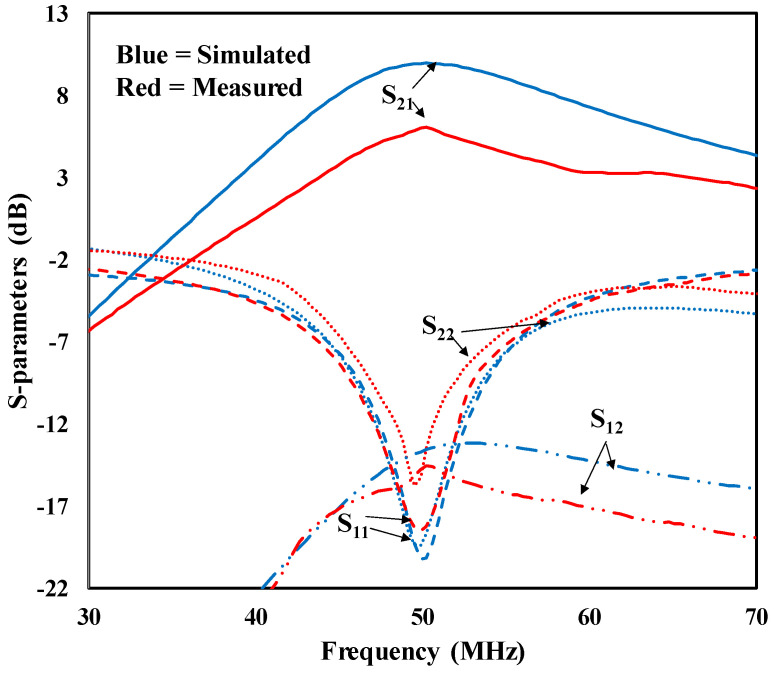
Measured and simulated S_11_, S_21_, S_22_ parameters for the amplifier circuit at 400 °C.

The S-parameters for the amplifier circuit were measured for frequencies between 20 and 100 MHz at temperatures between 25 °C and 400 °C using a precision network analyzer (Keysight N5224B). SOLT calibrations were performed to ensure measurement accuracy. Concurrently, simulated S-parameters were calculated for the amplifier model in ADS. Figure 17 shows the input return loss (S_11_), gain (S_21_), isolation (S_12_), and output return loss (S_22_) at 400 °C for both measured and simulated amplifier circuits. The plots are centered at the desired operating frequency of 50 MHz. The simulated input/output return losses of −20.1 dB and −19.5 dB compare favorably to the measured values of −18.5 and −15.2 dB. The simulated gain was 9.95 dB, which differs by only 3.94 dB from the measured gain, which was 6.01 dB. The simulated isolation was −13.65 dB, whereas the measured isolation was −14.48 dB. The difference between simulated and measured gain and isolation is due primarily to the ideal nature of a simulated environment, which provides conditions that are free of additional losses associated with such things as metallization and radiation. However, the S_12_ values demonstrate very good isolation between input and output ports.

**Figure 18:**
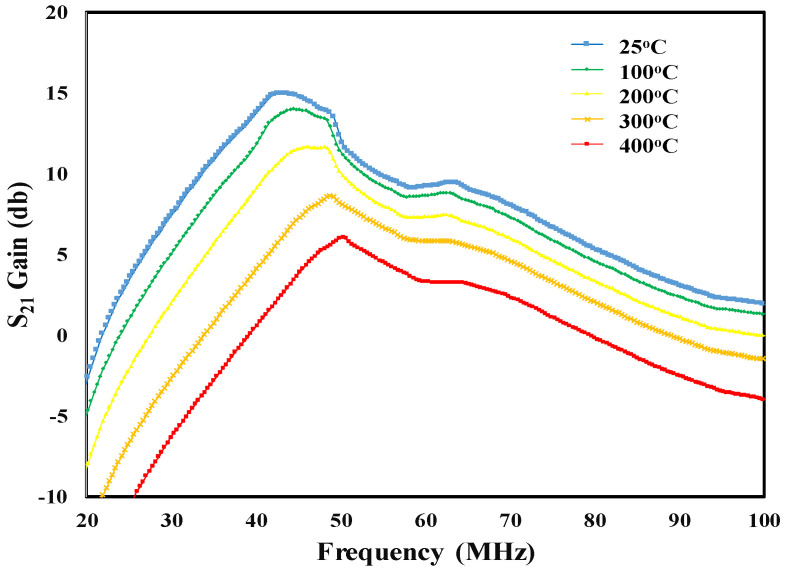
Amplifier gain (S_21_) as a function of frequency for temperatures.

Figure 18 presents the measured gain (S_21_) as a function of temperature for the fabricated amplifier circuit. This plot shows two temperature-related effects: (1) a decrease in gain with increasing temperature from 14.9 dB at 25 °C to 6.01 at 400 °C, and (2) a shift in the peak-gain from 43 MHz at 25 °C to 50 MHz at 400 °C. Regarding the former, previous work showed that the parasitic internal elements exhibit a temperature-dependent behavior, and if compensation for this temperature dependence is not implemented, the peak gain frequency of the amplifier would shift due to temperature effects [14]. Regarding the latter, the observed decrease in gain with increasing temperature can be attributed to a combination of effects, including a decrease in the transconductance and carrier mobility in the transistor, as well as an increase in the series resistances in the matching network. This effect is observed in the input/output spectrum of Figure 11 as well.

To evaluate the amplifier circuit for harmonic oscillations near the designed operating frequency of 50 MHz, the measured and simulated S-parameters were used to calculate the stability factor for frequencies between 30 and 70 MHz [17]. The stability factor, *K*, is calculated using

(2) $$K=\frac{1+\Delta^{2}-{\left|S_{11}\right|}^{2}-{\left|S_{22}\right|}^{2}}{2*\left|S_{12}S_{21}\right|}$$

where Δ is the determinant of the two-port S-parameter matrix, which can be found using

(3) $$\Delta =\left|S_{11}S_{22}-S_{12}S_{21}\right|$$

**Figure 19:**
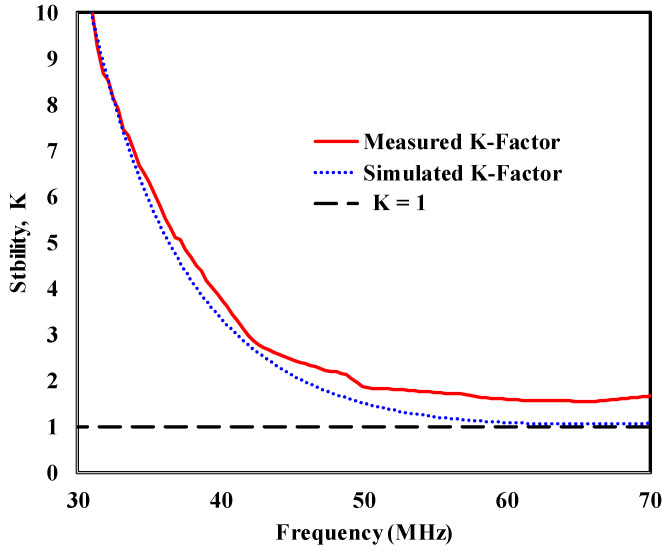
Measured and simulated stability factor (K) between 30 and 70 MHz at a temperature of 400 °C.

Figure 19 shows the stability, *K*, for the simulated and measured circuit at 400 °C. If the *K*-factor is greater than 1 and Δ is less than 1, the circuit is unconditionally stable and harmonic oscillations would not occur. Figure 19 shows that both measured and simulated circuits exhibit a stability factor greater than 1 over the entire frequency range, and therefore, the device is unconditionally stable over these frequencies. The K-factor at 50 MHz for the measured and simulated amplifiers are 1.86 and 1.50, respectively, a difference of only 24%. Figure 19 also shows that the measured stability is larger than the simulated stability for frequencies greater than ~35 MHz. This is likely due to the temperature-dependent increases in resistance caused by losses not accounted for in the circuit via wire bonds and transmission lines. This is consistent with the simulated and measured gain plotted in Figure 17, where the simulated gain is greater than the measured gain because of the parasitic resistances associated with metal lines and wire bond losses.

To further assess the stability of the amplifier circuit at 400 °C, singular two-port stability parameters, μ and μ′, were determined for both the simulated and fabricated circuits. For a two-port network such as the amplifier circuit developed in this work, this approach to evaluating stability provides a means to understand which side of the network would likely cause oscillations [18]. On the source side of the device, unconditional stability can be assessed by determining the stability parameter μ, which can be calculated from the S-parameters using the following formula.

(4) $$\mu =\frac{1-{\left|S_{11}\right|}^{2}}{\left|S_{22-}S{*}_{11}\Delta \right|+\left|S_{21}S_{12}\right|}$$

The input of a circuit is determined to be unconditionally stable when μ is greater than unity. On the output side of the circuit, μ′ is used to determine unconditional stability and can be calculated from the S-parameters using the following formula:

(5) $$\mu^{\prime }=\frac{1-{\left|S_{22}\right|}^{2}}{\left|S_{11-}S{*}_{22}\Delta \right|+\left|S_{21}S_{12}\right|}$$

If $\mu^{\prime }$ > 1, then the output is unconditionally stable. Unlike the K-factor, which only indicates whether the device as a whole is unconditionally stable, the μ-factors offer insight as to which side of the device would most likely experience instability.

**Figure 20:**
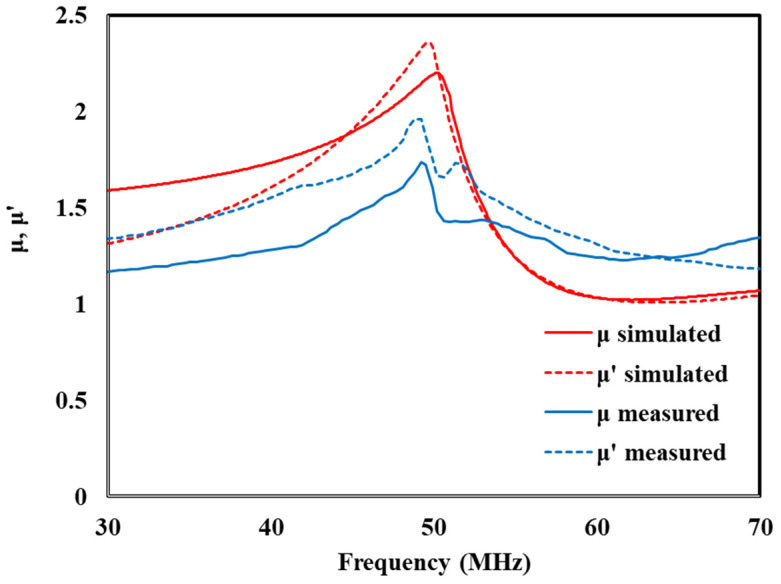
Two-port stability factors μ and μ′ for the fabricated and simulated amplifier at 400 °C for frequencies between 30 and 70 MHz.

Figure 20 presents plots of μ and μ′ for frequencies between 30 and 70 MHz. All μ and μ′ values for both the fabricated and simulated amplifier circuits exceed unity over this frequency range, indicating that the circuit is unconditionally stable on both input and output ports. It is observed that the μ and μ′ values for the simulated amplifier exhibit the same functional behavior with respect to frequency which indicates a similar level of stability. Likewise, the μ and μ′ values from the fabricated circuit also exhibit a similar functional behavior for frequencies up to ~60 MHz. Other than the clear offsets between the μ and μ′ plots for both simulated and measured circuits, the most significant difference between the μ and μ′ plots is the presence of a secondary peak in the μ′ plot at ~52 MHz for the fabricated amplifier. This may be due to variations in the matching circuits caused by unaccounted interference of the thin-film spiral inductors and additional losses in the through-lines and wire bonds generated in the network. The overall offset between the simulated and measured μ and μ′ plots can be attributed to these losses as well.

## 6. Conclusions

A SiC SIT-based Class-A amplifier that exhibits stable operation at 50 MHz and 400 °C was successfully fabricated, tested and modeled. The capacitors, inductors and resistors required for this amplifier exhibit stable operation up to 400 °C when tested as individual components. The S-parameters measured from the fabricated amplifier circuit show excellent agreement with the corresponding S-parameters of the circuit modeled in ADS at 400 °C. In particular, the differences in S_11_ and S_22_ values between the simulated amplifier and the fabricated amplifier at 50 MHz were only 1.6 dB and 4.3 dB respectively. Likewise, a difference in gain (S_21_) between the fabricated and simulated amplifier at 50 MHz was only 3.89 dB. The output power versus frequency spectra showed a frequency shift in the peak gain as a function of temperature, as well as a gain loss attributed to loss of carrier mobility in the transistor, increased resistances in the matching network, and an overall reduction in transconductance. The 1 dB compression was found to be at an input power of ~4 dBm, giving an upper operational input limit for the amplifier to function as a linear device. The noise figure indicated typical power amplifier behavior, especially at high temperatures, and the phase noise measurements indicated that there was not an appreciable amount of added phase noise at the input at a carrier frequency of 50 MHz. At 400 °C, the K-factor and the μ-factors show that the circuit exhibits stable operation between 30 and 70 MHz. In aggregate, these findings indicate that the high-frequency SiC SIT-based Class-A amplifier described in this paper exhibits satisfactory performance at 400 °C, and the simulation model that was developed to properly design the amplifier accurately predicts its performance. With a measured gain of 6 dB at 50 MHz, this simple amplifier is suitable for a wide range of wireless, harsh environment microsensor systems that require signal amplification at the point-of-use in a packaged system with a small form factor.

## Figures and Tables

**Table 1:** Component values of the Class-A amplifier generated by the optimized ADS model.

Component	Value
R_bias_	10 kΩ
L_bias_	400 nH
L_1_	95 nH
L_2_	100 nH
L_3_	105 nH
C_1_	270 pF
C_2_	270 pF
C_3_	50 pF

**Table 2:** Circuit model component values for the 94 pF capacitor for temperatures between 25 °C and 400 °C.

Temperature (°C)	Ls (pH)	Rs (mΩ)	Cs (pF)	Cp1 (fF)	Cp2 (pF)
25	0.30	320.23	94.00	303.01	1.52
100	0.30	351.37	94.04	303.01	1.44
200	0.31	383.84	94.00	313.03	1.52
300	0.33	402.35	95.50	333.03	1.52
400	0.35	424.24	96.45	315.23	1.52

**Table 3:** Circuit model component values for the 196 pF capacitor for temperatures between 25 °C and 400 °C.

Temperature (°C)	Ls (pH)	Rs (mOhm)	Cs (pF)	Cp1 (pF)	Cp2 (pF)
25	393.94	606.06	194.01	1.52	1.52
100	401.39	635.16	195.44	1.52	1.52
200	407.53	642.36	195.27	1.52	1.52
300	409.09	651.52	196.26	1.52	1.52
400	424.25	696.97	197.29	1.52	1.52

**Table 4:** Circuit model component values for the 94 nH spiral inductor for temperatures between 25 °C and 400 °C.

Temperature (°C)	Ls (nH)	Rs (Ohm)	Cs (pF)	Cp1 (fF)	Cp2 (fF)
25	94.17	3.74	0.88	0.53	0.29
100	94.22	4.56	0.88	0.53	0.29
200	94.56	6.14	0.88	0.53	0.29
300	95.11	9.55	0.88	0.53	0.29
400	95.86	12.87	0.88	0.53	0.29

## Data Availability

The datasets presented in this article are not readily available because the data is part of an ongoing study or but also is owned and maintained by the U.S. Government. Requests to access the datasets should be directed to Maximilian Scardelletti (maximilian.c.scardelletti@nasa.gov).

## References

[1] Hunter G.W., Simon D.L., Xu J.C., Biaggi-Labiosa A.M., Carranza S., Makel D.B. Aircraft Ground Demonstration of Engine Emissions Monitoring System based on a Gas Microsensor Array. Proceedings of the AIAA Joint Propulsion Conference.

[2] Meredith R.D., Wrbanek J.D., Fralick G.C., Greer L.C., Hunter G.W., Chen L. Design and operation of a fast, thin-film thermocouple probe on a turbine engine. Proceedings of the AIAA Joint Propulsion Conference.

[3] Woike M.R., Roeder J.W., Hughes C.E., Bencic T.J. Testing of a Microwave Blade Tip Clearance Sensor at the NASA Glenn Research Center. Proceedings of the AIAA Aerospace Science Mtg.

[4] Scardelletti M.C., Jordan J.L., Meredith R.D., Harsh K., Pilant E., Ursey M.W., Beheim G.M., Hunter G.W., Zorman C.A. Demonstration of a Packaged Capacitive Pressure Sensor System Suitable for Jet Turbofan Engine Health Monitoring. Proceedings of the 2016 IEEE Electronics and Components Technology Conference.

[5] Wang R., Ko W.H., Young D.J. (2005). Silicon-carbide MESFET-cased 400 °C sensing and data telemetry. IEEE Sens. J..

[6] Yang J. (2013). A Harsh Environment Wireless Pressure Sensing Solution Utilizing High Temperature Electronics. Sensors.

[7] Scardelletti M. (2016). Development of a High Temperature Silicon Carbide Capacitive Pressure Sensor System Based on a Clapp-Type Oscillator Circuit. Ph.D. Dissertation.

[8] Scardelletti M.C., Ponchak G.E., Harsh K., Mackey J.A., Meredith R.D., Zorman C.A., Beheim G.M., Dynys F.W., Hunter G.W. Wireless capacitive pressure sensor operating up to 400 °C from 0 to 100 psi utilizing power scavenging. Proceedings of the 2014 IEEE Topical Conference on Wireless Sensors and Sensor Networks (WiSNet).

[9] Neudeck P.G., Okojie R.S., Chen L. (2002). High-temperature electronics—A role for wide bandgap semiconductors?. Proc. IEEE.

[10] Sheppard S.T., Slater D.B., Lipkin L.A., Das M.K., Suvorov A.V., Palmour J.W. High Temperature Demonstration of a CMOS Operational Amplifier using 6H Silicon Carbide N-Well Technology and ONO Dielectrics. Proceedings of the 5th International High Temperature Electronics Conference (HiTEC).

[11] Patil A.C., Fu X.A., Mehregany M., Garverick S.L. Fully-monolithic, 600 °C differential amplifiers in 6H-SiC JFET IC technology. Proceedings of the 2009 IEEE Custom Integrated Circuits Conference.

[12] Hedayati R., Lanni L., Rodriguez S., Malm B.G., Rusu A., Zetterling C. (2014). A Monolithic, 500 °C Operational Amplifier in 4H-SiC Bipolar Technology. IEEE Electron Device Lett..

[13] Hiemstra R. (2015). A High Temperature Wideband Power Amplifier for a Downhole Communication System. Master’s Thesis.

[14] Grgat J.R., Scardelletti M.C., Zorman C.A. (2025). Evaluation of a Silicon Carbide Static Induction Transistor for High Frequency/High Temperature Sensor Interface Circuits: Measurements and Modeling. Sensors.

[15] Advanced Design System. https://www.keysight.com/us/en/home.html.

[16] Schwartz Z.D., Downey A.N., Alterovitz S.A., Ponchak G.E. High-temperature probe station for use in microwave device characterization through 500 °C. Proceedings of the 61st ARFTG Conference Digest, Spring 2003.

[17] Gonzalez G. (1996). Microwave Transistor Amplifiers: Analysis and Design.

[18] Edwards M.L., Sinsky J.H. (1992). A new criterion for linear 2-port stability using a single geometrically derived parameter. IEEE Trans. Microw. Theory Tech..

